# Uterine cancer deaths certified as part unspecified: an unsolved issue

**DOI:** 10.1097/CEJ.0000000000000833

**Published:** 2023-08-17

**Authors:** Giovanna Esposito, Claudia Santucci, Fabio Parazzini, Silvia Mignozzi, Matteo Malvezzi, Carlo La Vecchia, Eva Negri

**Affiliations:** a Department of Clinical Sciences and Community Health, University of Milan, Milan; b Department of Medicine and Surgery, University of Parma, Parma; c Department of Medical and Surgical Sciences, University of Bologna, Bologna, Italy

**Keywords:** cervical cancer, endometrial cancer, mortality, unspecified uterine cancer

## Abstract

**Objective:**

A large percentage of uterine cancer deaths worldwide are not attributed to the cervix or corpus, but classified as uterus part ‘unspecified’. We provided the trend for the proportion of uterine cancer deaths certified as ‘unspecified’ in selected countries.

**Methods:**

We derived the proportions of ‘unspecified’ uterine cancers for 20 selected high- and middle-income countries with reliable death certification over the period 1994–2021, using official mortality data from the WHO database coded according to the 10th Revision of the International Classification of Diseases.

**Results:**

For the earliest available year, the proportion of deaths classified as ‘unspecified’ uterine cancers ranged from 5.8% in Mexico to 65.6% in Italy. In some countries only, this proportion decreased over time. For 10 countries the proportion of ‘unspecified’ uterus in the most recent available year was around 20%. The proportion of deaths at 20–44 years registered as uterus ‘unspecified’ was lower for all countries during the study period.

**Conclusion:**

A substantial number of uterine cancer deaths worldwide coded as ‘unspecified’ was observed, also in high-income countries where death certification for other common neoplasms is accurate. Valid attribution of uterine cancer deaths to the cervix or corpus is feasible and should be adopted.

## Introduction

The analysis of mortality data from cancers of the cervix and corpus uteri using routine sources is hindered by inaccuracy in death certificates. A large proportion of uterine cancer deaths are registered as ‘malignant neoplasm of uterus, part unspecified’. The proportion of these deaths in relation to all uterine cancer mortality varies greatly worldwide and also some high-income countries code over 50% of uterine cancer deaths as ‘unspecified’ (Loos *et al*., 2004).

Cervical and uterine corpus (mainly endometrial) cancers are distinct malignancies, differing in terms of their anatomical localization and also their epidemiological characteristics, etiology, management, and prognosis (Parazzini *et al*., 1998; Shi *et al*., 2023). Therefore, it is crucial to consider them separately.

Analyzing temporal trends in cervical uterine cancer mortality helps in assessing the effectiveness of preventive measures such as regular screening programs and human papillomavirus vaccination, which are known to prevent the disease.

The issue of ‘unspecified’ uterine cancer deaths was addressed in different ways throughout the literature (Sanchez Garrido *et al*., 1996; Arbyn and Geys, 2002; Loos *et al*., 2004; Partanen *et al*., 2022). However, no method has been satisfactory. We therefore provided a global overview regarding this coding issue, focusing mainly on high- and middle-income countries where high data coverage and accuracy are expected and attainable. In this perspective, we showed the trend for the proportion of uterine cancer deaths certified as ‘unspecified’ in selected countries to determine whether additional efforts are required to optimize the collection of data from death certificates.

## Materials and methods

We retrieved official mortality data on uterine cancer from the WHO database, starting from the introduction of the 10th Revision of the International Classification of Diseases up to the latest available year. We focused on the specific codes for malignant neoplasm of the corpus uteri (C53), cervix uteri (C54), and uterus part unspecified (C55). For each year and country, we obtained the proportion of ‘unspecified’ uterine cancer deaths. We restricted our report to 20 most populous countries (i.e. over 10 million for Europe and over 25 million for all other countries), with high data coverage, and high data quality as declared by the WHO. These include 16 high-income (i.e. Belgium, the Czech Republic, France, Germany, Italy, the Netherlands, Portugal, Romania, Spain, Sweden, the UK, Canada, the USA, Chile, Japan, and Australia) and 4 middle-income (i.e. Argentina, Brazil, Colombia, and Mexico) countries.

## Results

**Fig. 1:**
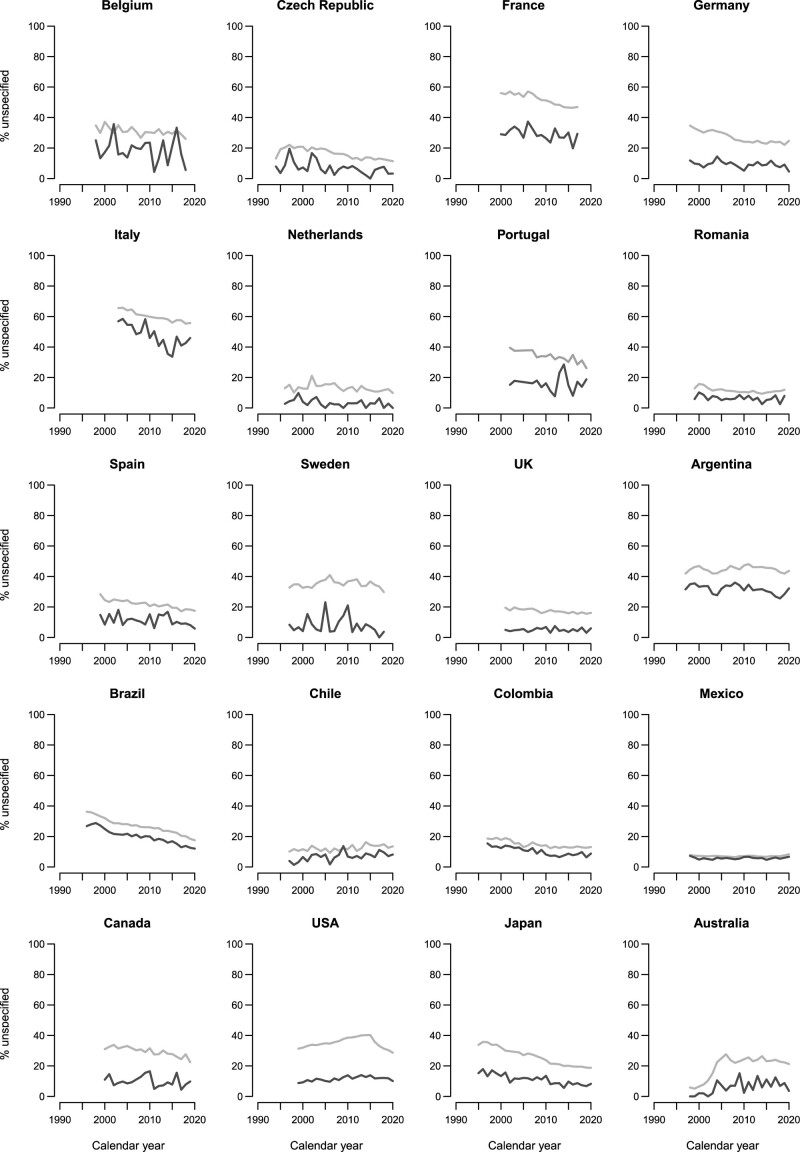
Trends in the proportion of uterine cancer deaths certified as 'unspecified' in selected countries (1994-2021) at all ages (grey) and at 20-44 years (dark grey).

Figure 1 illustrates the trend in the proportions of uterine cancer deaths certified as ‘unspecified’ for selected countries between 1994 and 2021 at all ages and at 20–44 years. Table 1 shows these proportions in the first and last available year.

**Table 1:** The proportion of uterine cancer deaths certified as ‘unspecified’ in selected countries in the first and last available year at all ages and at 20–44 years

Country	Calendar period	% ‘unspecified’			
		First available year		Last available year	
		All ages	20–44 years	All ages	20–44 years
Europe					
Belgium	1998–2018	34.8	25.0	26.1	5.6
Czech Republic	1994–2021	13.1	7.9	12.5	9.4
France	2000–2017	56.0	29.0	46.9	29.3
Germany	1998–2020	34.7	11.8	24.7	4.6
Italy	2003–2019	65.6	56.9	55.8	45.9
Netherlands	1996–2020	13.0	2.8	9.9	0.0
Portugal	2002–2019	39.5	15.2	26.2	18.8
Romania	1999–2019	12.9	5.8	11.9	7.9
Spain	1999–2021	28.4	14.8	18.2	8.3
Sweden	1997–2018	32.7	8.3	29.8	3.7
UK	2001–2020	19.5	5.0	16.1	6.0
North America					
Canada	2000–2019	31.0	11.0	22.5	9.8
USA	1999–2020	31.4	8.8	28.6	10.1
Latin America					
Argentina	1997–2020	42.0	31.5	43.7	32.2
Brazil	1996–2020	36.2	26.8	17.5	12.1
Chile	1997–2020	10.1	3.9	13.5	8.2
Colombia	1997–2020	18.6	15.4	13.1	8.8
Mexico	1998–2020	7.8	7.3	8.3	6.7
Australasia					
Australia	1998–2021	5.8	0.0	21.2	6.7
Japan	1995–2020	33.9	15.3	18.8	8.2

The country with the lowest proportion of ‘unspecified’ uterine cancer deaths was Mexico with less than 10% over the time period considered. For Chile, the Netherlands, the Czech Republic, and Romania, the proportion was slightly over 10%. No trend emerged for these countries. Italy had the highest proportion, exceeding 60% at the beginning of the 2000s. France and Argentina also had more than 40% of ‘unspecified’ uterine cancer deaths. Italian proportions moderately decreased from 65.6% in 2003 to 46.9% in 2019 while in France they remained approximately stable at 56.0% in 2000 and 55.8% in 2017.

In other European countries analyzed, that is, Belgium, Germany, Portugal, and Sweden, the proportion of ‘unspecified’ uterus ranged from 20 to 40%. Spain and the UK registered proportions of around 20% or slightly lower. For all these countries, the proportion of ‘unspecified’ decreased over time.

In North America, ‘unspecified’ uterine cancer deaths were approximately 30%. In the USA, this percentage increased up to 2015 with a peak of 40.0% and then decreased. In Latin America, Colombia accounted for less than 1 out of 5 uterine cancer deaths classified as ‘unspecified’ during the considered time period, whereas in Brazil the proportion of ‘unspecified’ decreased from 36.2% in 1996 to 17.5% in 2020.

Declines were also observed in Japan, from 33.9% in 1995 to 18.8% in 2020. In contrast, in Australia, the number of ‘unspecified’ uterine cancer deaths increased from 5.8% in 2000 to around 20.0% in 2005 and remained stable thereafter.

The proportion of deaths at 20–44 years registered as uterus ‘unspecified’ was lower for all countries during the study period. However, in some countries, such as France, Italy, and Argentina, the proportion was still above 20.0% in the last available year.

## Discussion

Even over the most recent calendar years, a substantial number of uterine cancer deaths worldwide are coded as ‘unspecified’ without distinction between the cervix and corpus. The proportion of these deaths in relation to all uterine cancer deaths varied considerably, the highest was observed in Italy and the lowest in Mexico. It is known that cancer death certification is more valid among young than elderly women (Doll and Peto, 1981). However, in several countries, including France, Italy, and Argentina, a considerable proportion of deaths at 20–44 years was registered as uterus ‘unspecified’.

The large number of uterine cancer deaths coded as ‘malignant neoplasm of uterus, part unspecified’ is a persistent unresolved question, partly due to an inadequate training of physicians completing death certificates (Arbyn and Geys, 2002) and partly associated with possible stigma related to a diagnosis of cervical cancer. This cannot be attributed to diagnostic difficulties since the distinction between cervical and endometrial cancer is not complex.

Although methods have been developed to allocate deaths classified as ‘unspecified’ to either the cervix or corpus uteri, they have not proven adequate in addressing this issue except by restricting analyses to younger women (below age 45 years) when most cancers arise for the cervix (Wojtyla *et al*., 2020). Reallocation based on age- and time-specific distributions of cervix and corpus uteri cancer were proposed and used for data from several countries (Loos *et al*., 2004; Partanen *et al*., 2022). For example, in a study involving European women, the analysis was restricted to the age group 20–44 years, assuming that the majority or almost the totality of deaths were due to cervical cancer (Levi *et al*., 2000). Others analyzed only certified cervical cancer, combining unspecified with corpus uteri cancers (Sanchez Garrido *et al*., 1996).

In the late 1970s in the USA, 25% of uterine cancer deaths were not specified as either cervix or corpus uteri on death certificates; comparing the causes of death listed on the death certificates with hospital and pathologic diagnoses, the majority of deaths were attributed to corpus uteri (Percy *et al*., 1983). This is not surprising, since endometrial cancer is common in older age, when the certification is less accurate. A similar approach was also adopted for data from Catalonia through the linkage with the Cancer Registry of Girona, finding that one-third of ‘unspecified’ uterus cancer in death certificates arose in the cervix, one-third in the corpus, and one-third remained ‘unspecified’ (Sanchez Garrido *et al*., 1996).

Differentiating between mortality data for corpus and cervix cancer provides understanding of their contributions to mortality, as well as insights into treatment outcomes, survival rates, and potential gaps in care for cervical and endometrial cancers. Additionally, this would offer valuable information to identify high-risk populations, developing targeted prevention strategies, and allocating resources.

In conclusion, the unsolved issue of accurately defining the site of uterine tumors, cervix or corpus, has implications for research, public health initiatives, and clinical practice. Further efforts are needed to reduce stigma and ensure that mortality data reflect the true distribution and impact of uterine cancer subtypes.

## Acknowledgements

This work was supported by the Italian Association for Cancer Research (AIRC, project N. 22987). In addition, Dr C.S. was supported by EU funding within the Next Generation EU-MUR PNRR Extended Partnership initiative on Emerging Infectious Diseases (Project no. PE00000007, INF-ACT).

### Conflicts of interest

There are no conflicts of interest.
